# Supplementary material for: The lncRNA SNHG26 drives the inflammatory-to-proliferative state transition of keratinocyte progenitor cells during wound healing
Source: Nat Commun. 2024 Oct 5;15:8637. doi: 10.1038/s41467-024-52783-8 (PMC11452505; doi:10.1038/s41467-024-52783-8)
Supplement: Supplementary file 1 — Supplementary Information [file 41467_2024_52783_MOESM1_ESM.pdf]

# Supplementary Information

## The lncRNA *SNHG26* drives the inflammatory-to-proliferative state transition of keratinocyte progenitor cells during wound healing

Dongqing Li[1,*], Zhuang Liu[2,6], Letian Zhang[2,6], Xiaowei Bian[2,6], Jianmin Wu[3], Li Li[1], Yongjian Chen[2], Lihua Luo[2], Ling Pan[1], Lingzhuo Kong[1], Yunting Xiao[1], Jiating Wang[1], Xiya Zhang[1], Wang Wang[4], Maria Toma[2], Minna Piipponen[2], Pehr Sommar[5], Ning Xu Landén[2,*]

[1]Key Laboratory of Basic and Translational Research on Immune-Mediated Skin Diseases, Chinese Academy of Medical Sciences; Jiangsu Key Laboratory of Molecular Biology for Skin Diseases and STIs; Institute of Dermatology, Chinese Academy of Medical Sciences and Peking Union Medical College, 210042 Nanjing, China.

[2]Dermatology and Venereology Division, Department of Medicine Solna, Center for Molecular Medicine, Karolinska Institutet, 17176 Stockholm, Sweden.

[3]Institute of Genomic Medicine, Wenzhou Medical University, 325035 Wenzhou, China.

[4]Shanghai Key Laboratory of Regulatory Biology, School of Life Sciences, East China Normal University, 200241 Shanghai, China.

[5]Department of Plastic and Reconstructive Surgery, Karolinska University Hospital, 17176 Stockholm, Sweden.

[6]These authors contribute equally to this work.

* Correspondence: ning.xu@ki.se (N.X.L.) and dongqing.li@pumcderm.cams.cn (D.L.)

**Supplementary Figure 1.**

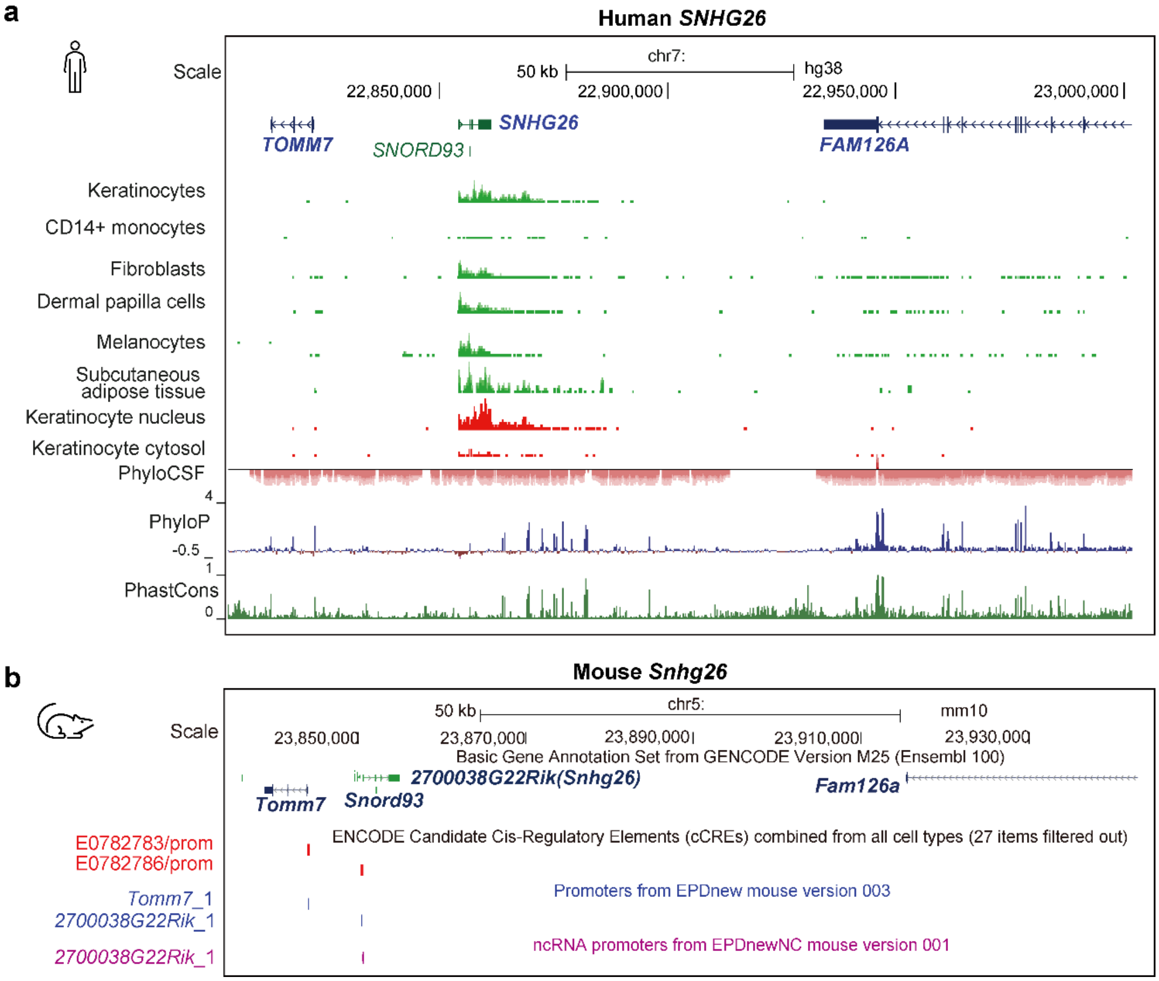


**Supplementary Figure 1. The genomic locus of SNHG26 in human and mouse during skin wound healing.** (**a**) UCSC genome browser snapshot of *SNHG26* region and its neighborhood genes generated in GENECODE V38. Data were retrieved from Encyclopedia of DNA Elements data hub, phylogenetic information-based codon substitution frequency (PhyloCSF), and conservation tracks (PhyloP and PhastCons). RNA-seq data of *SNHG26* in different cell types isolated from human skin and in the cytoplasmic and nuclear fractions of human keratinocyte progenitors were shown. (**b**) UCSC genome browser snapshot of mouse *Snhg26* (*2700038G22Rik*). Data were retrieved from ENCODE candidate Cis-regulatory element (cCREs) combined from all cell types, promoters from Eukaryotic promoter database (EPD).

## Supplementary Figure 2.

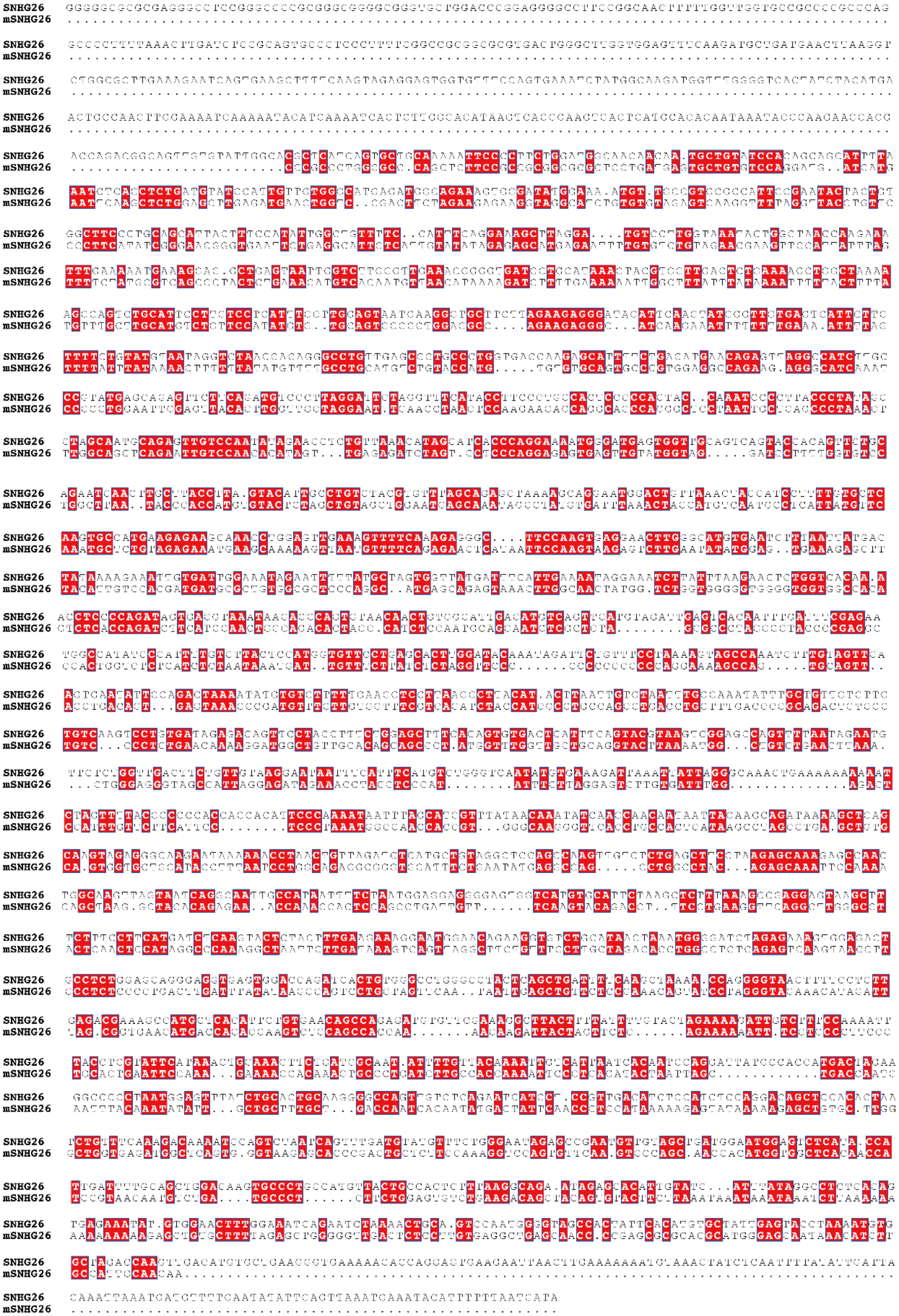


**Supplementary Figure 2. RNA sequence comparison of human and mouse SNHG26.**

**Supplementary Figure 3.**

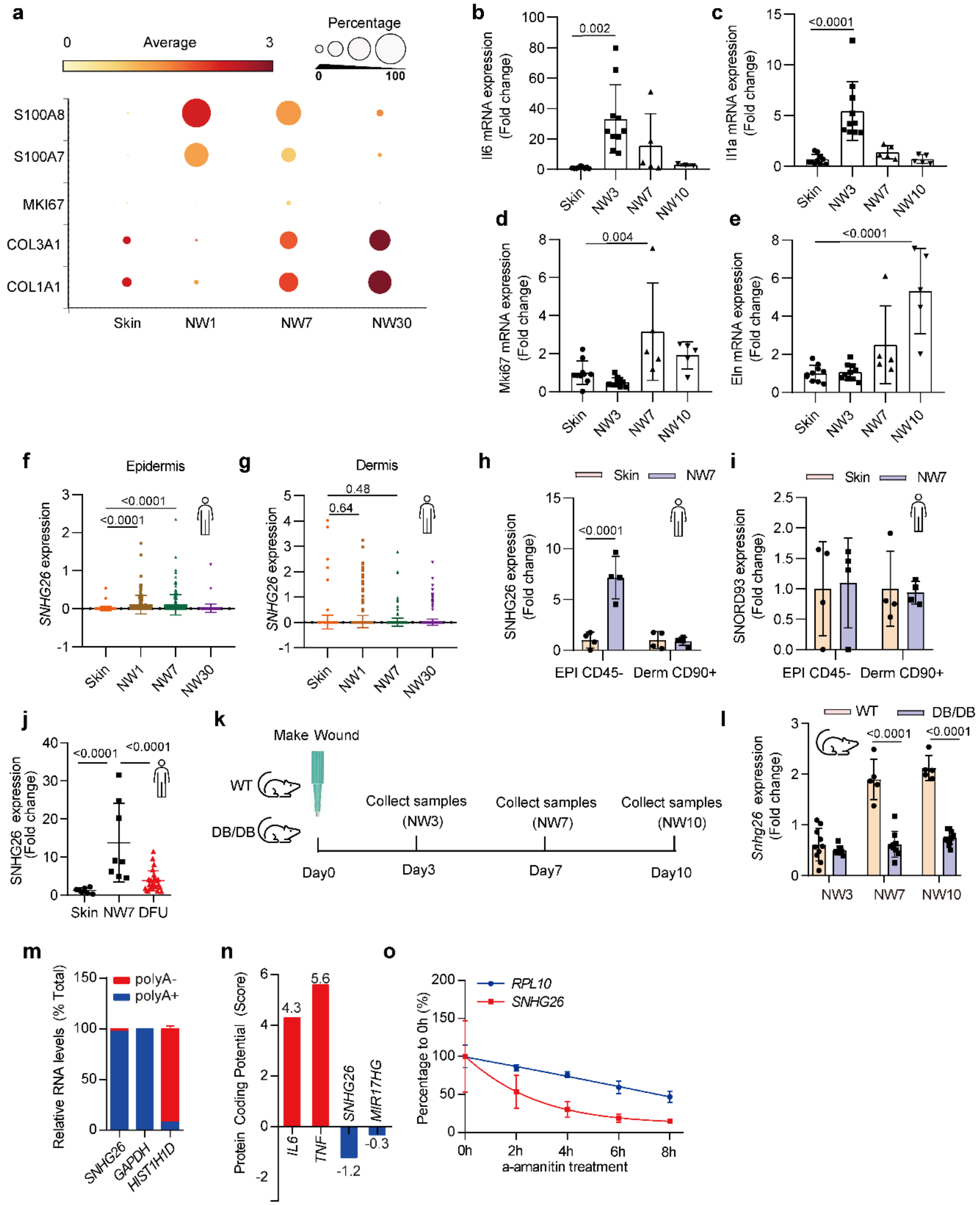


**Supplementary Figure 3. The expression dynamics of SNHG26 in human and mouse during skin wound healing.** (**a**) The expression of *S100A7, S100A8, MKI67, COL1A1*, and *COL3A1* in single cell RNA sequencing data of human acute wounds. (**b-e**) qRT-PCR analysis of *Il6, Il1a, Mki67* and *Eln* in murine acute wounds (n=5-10). (**f-g**) The expression of *SNHG26* in the epidermis

(**f**) and dermis (**g**) of human skin and acute wounds analyzed by spatial transcriptomics. (**h-i**) RNA sequencing analysis of *SNHG26* (**h**) and *SNORD93* expression (**i**) in epidermal keratinocytes (EPI CD45-) and dermal fibroblasts (Derm CD90+) isolated from matched human skin and day-7 acute wounds (NW7) (n=5 donors). (**j**) qRT–PCR analysis of SNHG26 expression in human skin, NW7 (n= 8 healthy donors), and diabetic foot ulcers (DFU, n= 29). (**k**) 4-mm wounds were made on the WT and DB/DB mice back skin, Skin biopsies at the wound edge and intact area were collected 3,7 and 10 days after injury. (**l**) qRT–PCR analysis of *Snhg26* expression in the wounds of db/db mice and wild type mice (n= 5-10). (**m**) qRT–PCR analysis of *SNHG26*, *GAPDH*, and *HIST1H1D* in poly(A)+ and poly(A)− RNA fractions from human keratinocyte progenitors (n=3). (**n**) Protein-coding potential analysis of *IL6* mRNA, *TNF* mRNA, *SNHG26*, and *MIR17HG* RNA. (**o**) qRT–PCR analysis of *SNHG26* and *RPL10* mRNA in keratinocytes treated with α-amanitin (5 μg/ml) for 2-8 hours (n=3). Data are shown as mean ± SD from two to three independent experiments (**j-o**). The data were analyzed by One-way ANOVA (**b-g**, **j**) or Two-way ANOVA analysis (**h, l**).

**Supplementary Figure 4.**

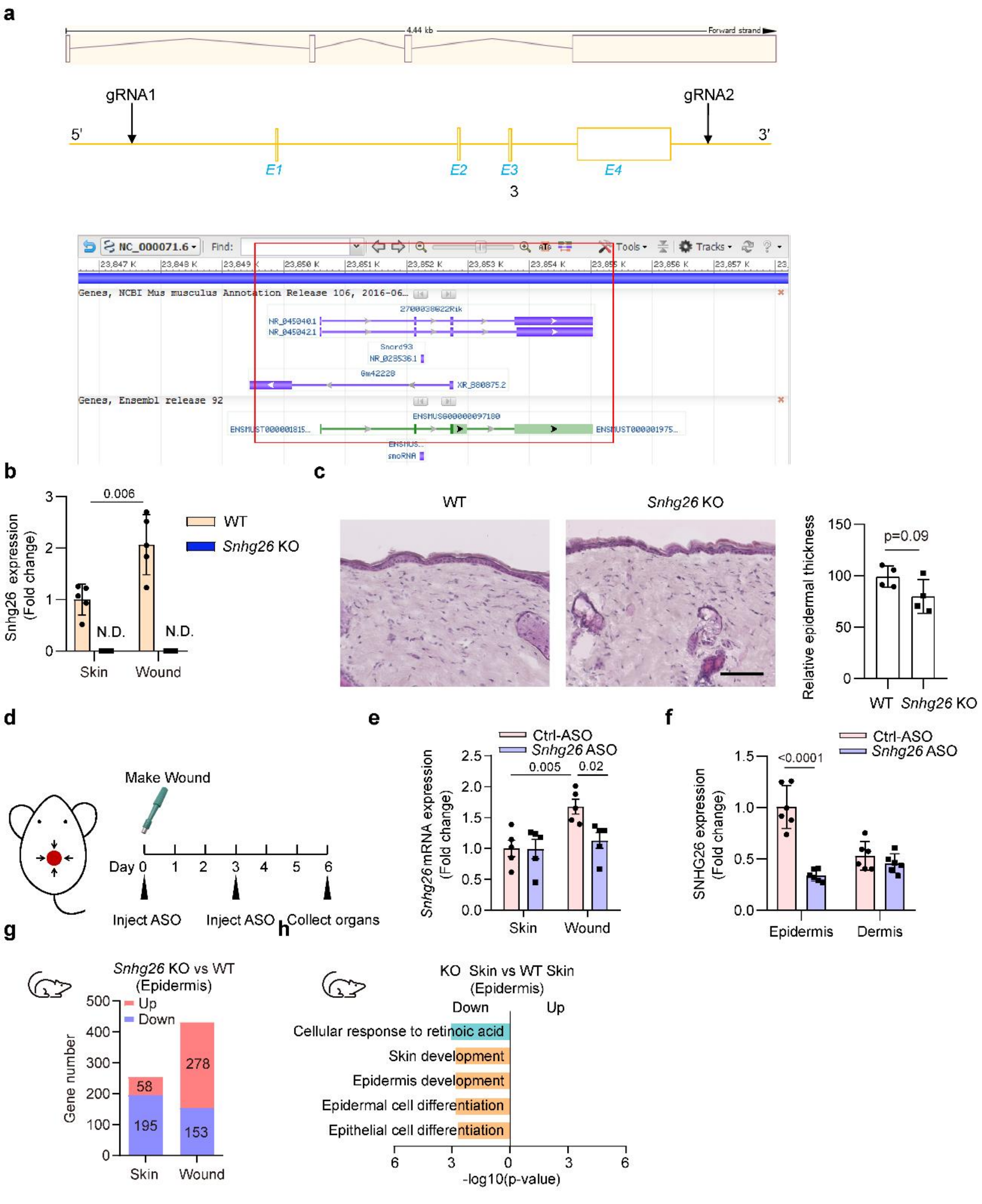


**Supplementary Figure 4. Impaired wound healing in *Snhg26*-deficient mice.** (**a**) Genomic snapshot of the *Snhg26* knockout (KO) area in mice. (**b**) qRT-PCR analysis of *Snhg26* expression in the skin and wounds of *Snhg26*-KO (n= 5) and wild-type (WT, n= 5) mice. n.d.: not detected. (**c**) Hematoxylin and Eosin staining of the skin from *Snhg26*-KO mice (n = 4) and WT mice (n = 4). The epidermis thickness was analyzed by Image J. Scale bars: 100 µm. (**d**) The workflow of *Snhg26*-ASO injection into the mice wounds. The skin and wound tissues were collected at Day 6.

(**e**) qRT-PCR analysis of the expression of *Snhg26* in the skin (n= 5) and wounds (n= 5) after *Snhg26*-ASO injection. (**f**) qRT-PCR analysis of the expression of *Snhg26* in the epidermis (n= 6) and dermis (n= 6) of day-6 wounds after *Snhg26*-ASO treatment. (**g**) The number of upregulated and downregulated genes (p value < 0.05, fold change ≥ 2 or ≤ 0.5) in the epidermis of skin and wounds from the *Snhg26*-KO mice compared to the WT mice, as shown in the microarray analysis. (**h**) GO analysis of the upregulated and downregulated genes in skin epidermis of the *Snhg26*-KO mice compared to the WT mice. Data are shown as mean ± SD from two to three independent experiments (**b-f**). The data were analyzed by two-way ANOVA (**b**, **e**, **f**) or two-tailed Student's t test (**c**).

**Supplementary Figure 5.**

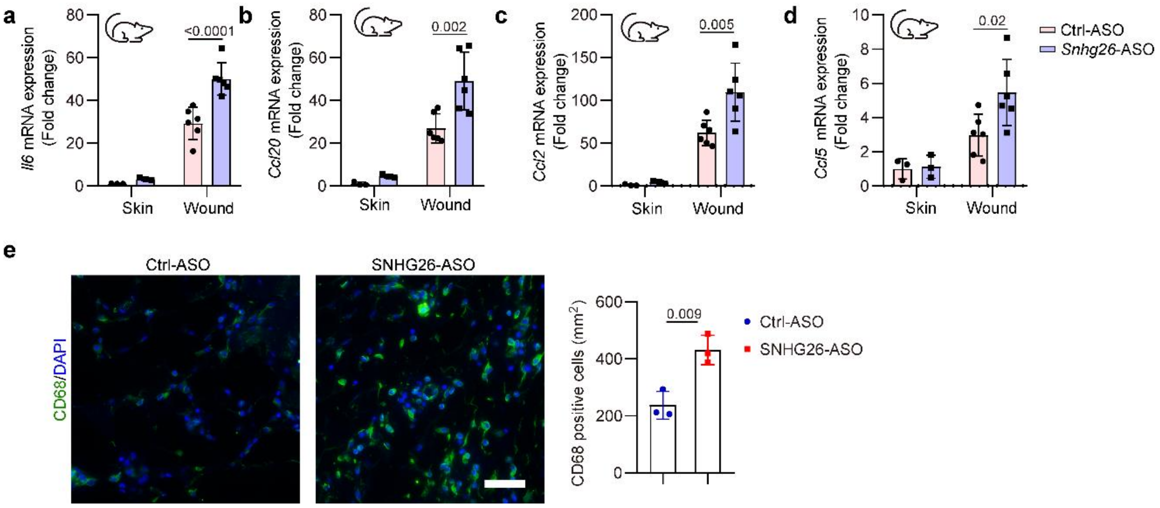


**Supplementary Figure 5. Increased inflammatory response in the wound of mice injected with SNHG2-ASO.** (**a-d**) qRT-PCR analysis of the expression of *Il6*, *Ccl20*, *Ccl2,* and *Ccl5* in the skin and the day-6 wounds after *Snhg26*-ASO treatment (n=3-6). (**e**) Immunofluorescence analysis of CD68+ macrophages in mouse wounds injected with SNHG26-ASO (n=3). Scale bar, 20 μm. Data are shown as mean ± SD from two to three independent experiments. The data were analyzed by two-way ANOVA (**a-d**) or two-tailed Student's t test (**e**).

**Supplementary Figure 6.**

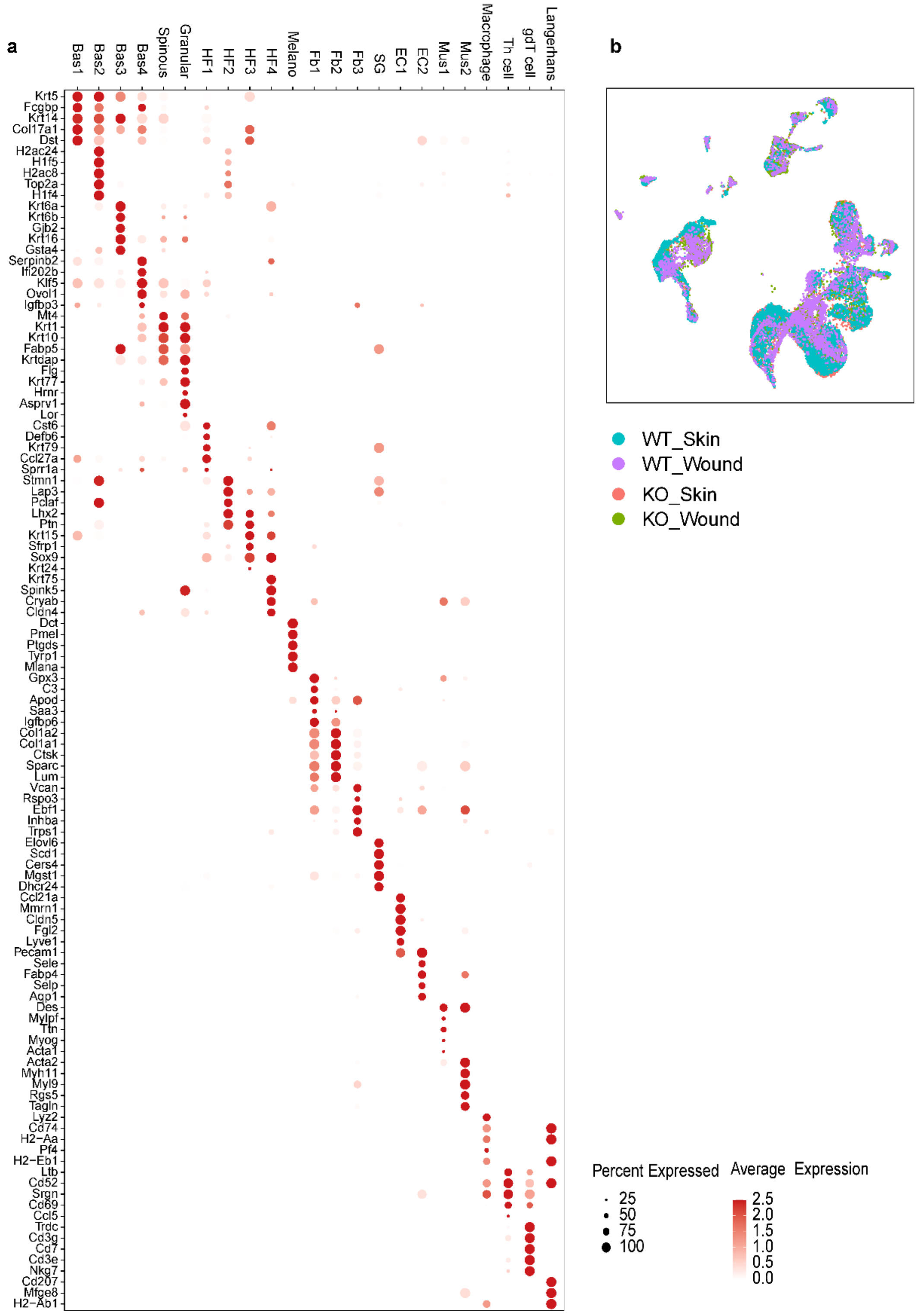


**Supplementary Figure 6. Single cell RNA sequencing (scRNA-seq) analysis of the skin and wound cells from *Snhg26*-KO and WT mice.** (**a**) Marker genes of each cell cluster in mice skin and wound. (**b**) UMAP projection of all cells sorted by sample types.

**Supplementary Figure 7.**

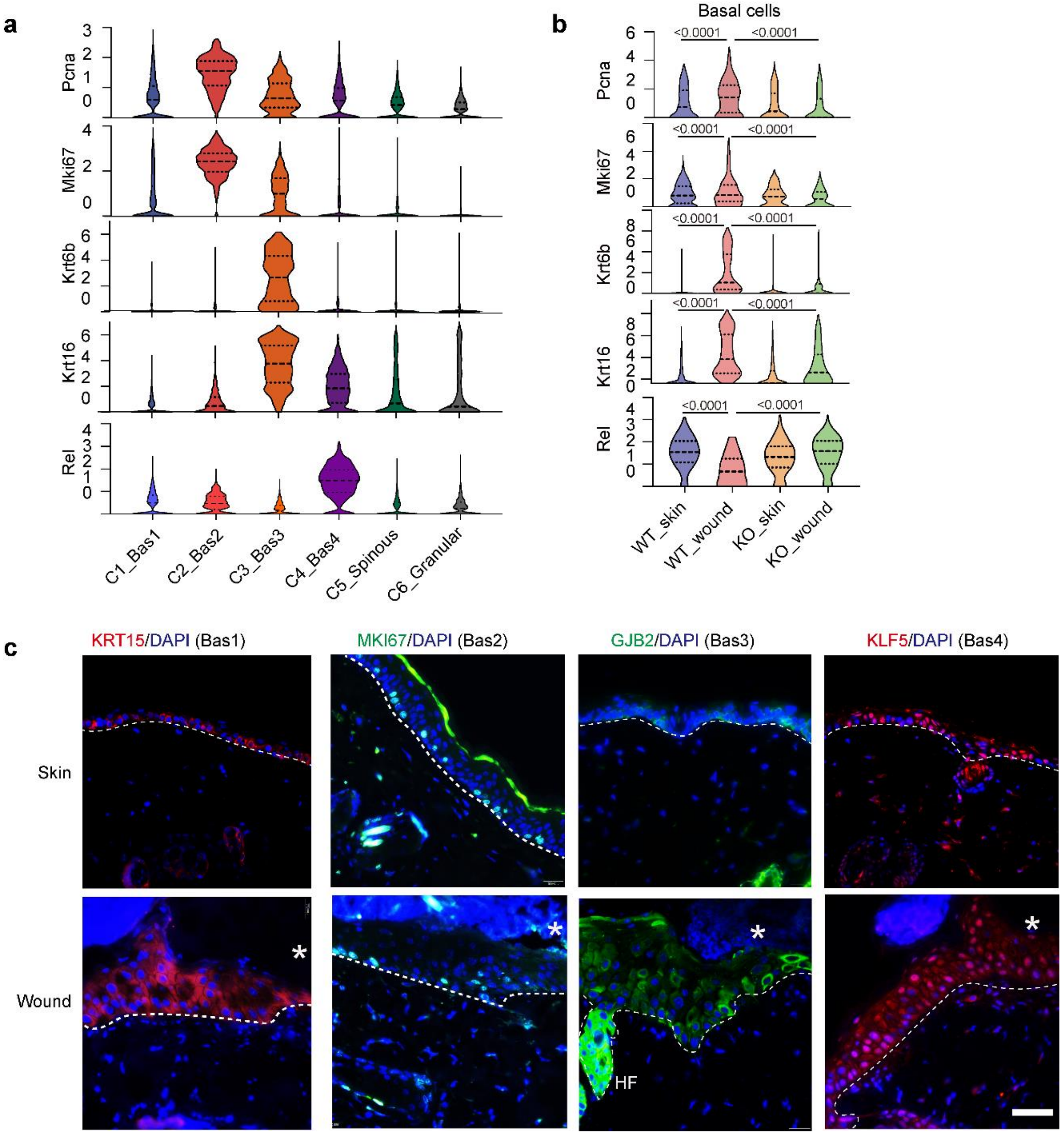


**Supplementary Figure 7. Marker genes of keratinocytes subpopulations in the skin and wound from *Snhg26*-KO and WT mice.** (**a-b**) Violin plots illustrating the expression of proliferation markers (*Pcna* and *Mki67*), migration markers (*Krt6b* and *Krt16*) and inflammatory genes (*Rel*) in six keratinocyte clusters (**a**), and in the basal keratinocytes from the skin and wounds of *Snhg26*-KO mice and WT mice (**b**). (**c**) Immunofluorescence staining of the marker genes of basal keratinocyte clusters in the skin and wound edge of wild-type mice. The asterisk (*) indicates the wound edge. HF: Hair follicle. Scale bar, 100 µm. The white star represents the wound edge. Scale bar, 20 µm. The data were analyzed one-way ANOVA (**b**).

**Supplementary Figure 8.**

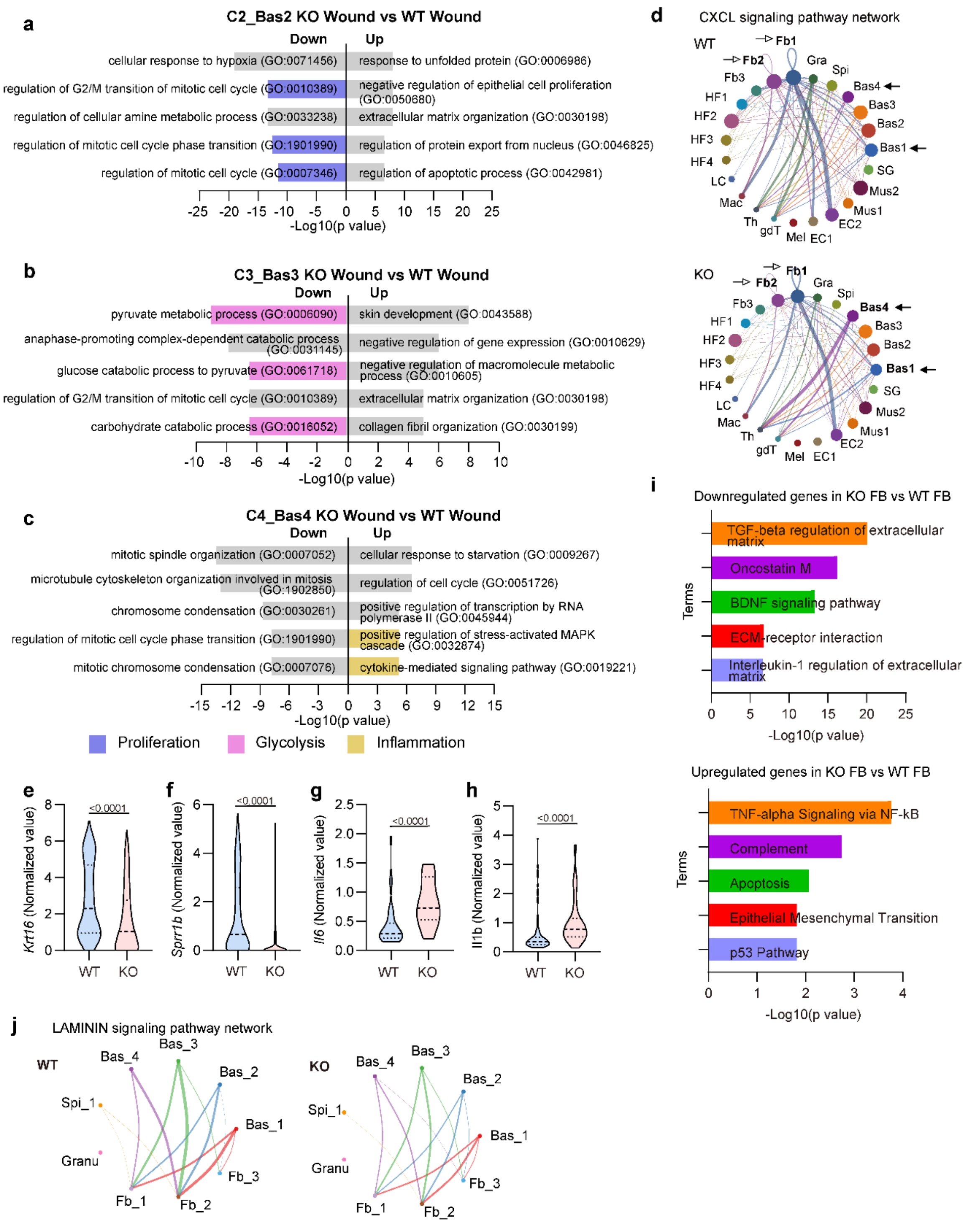


**Supplementary Figure 8. Cell-cell crosstalk and fibroblasts gene expression analysis with the scRNA-seq data of *Snhg26*-KO and WT mice.** (**a-c**) GO analysis of the top 300 up-and down-regulated genes in in the Bas2, Bas3 and Bas4 keratinocytes in wounds of the *Snhg26*-KO mice compared with the WT mice. GO terms related to proliferation, glycolysis, and inflammation were

highlighted. (**d**) Circle plots display the CXCL signaling network in *Snhg26*-KO and WT mice wounds. The edge width is proportional to the communication score between interacting cells. The changed signals in the KO mice compared to the WT mice were highlighted with arrows. (**e-h**) Violin plots showing the expression of *Krt16* (**e**), *Sppr1b* (**f**), *Il6* (**g**), and *Il1b* (**h**) in the wound edge keratinocytes of Snhg26-KO and WT mice analyzed by scRNA-seq. (**i**) Enrich R pathway analysis of the down- and up-regulated genes [$\log_2(FC) \geq 0.3$, $-\log_{10}(FDR) > 2$] in the fibroblasts of *Snhg26*-KO mice wound compared to WT mice wound. (**j**) Circle plots display the Laminin signaling network in *Snhg26*-KO and WT mice wounds. The edge width is proportional to the communication score between interacting cells. The data were analyzed by two-sided Mann-Whitney U test (**e-h**).

**Supplementary Figure 9.**

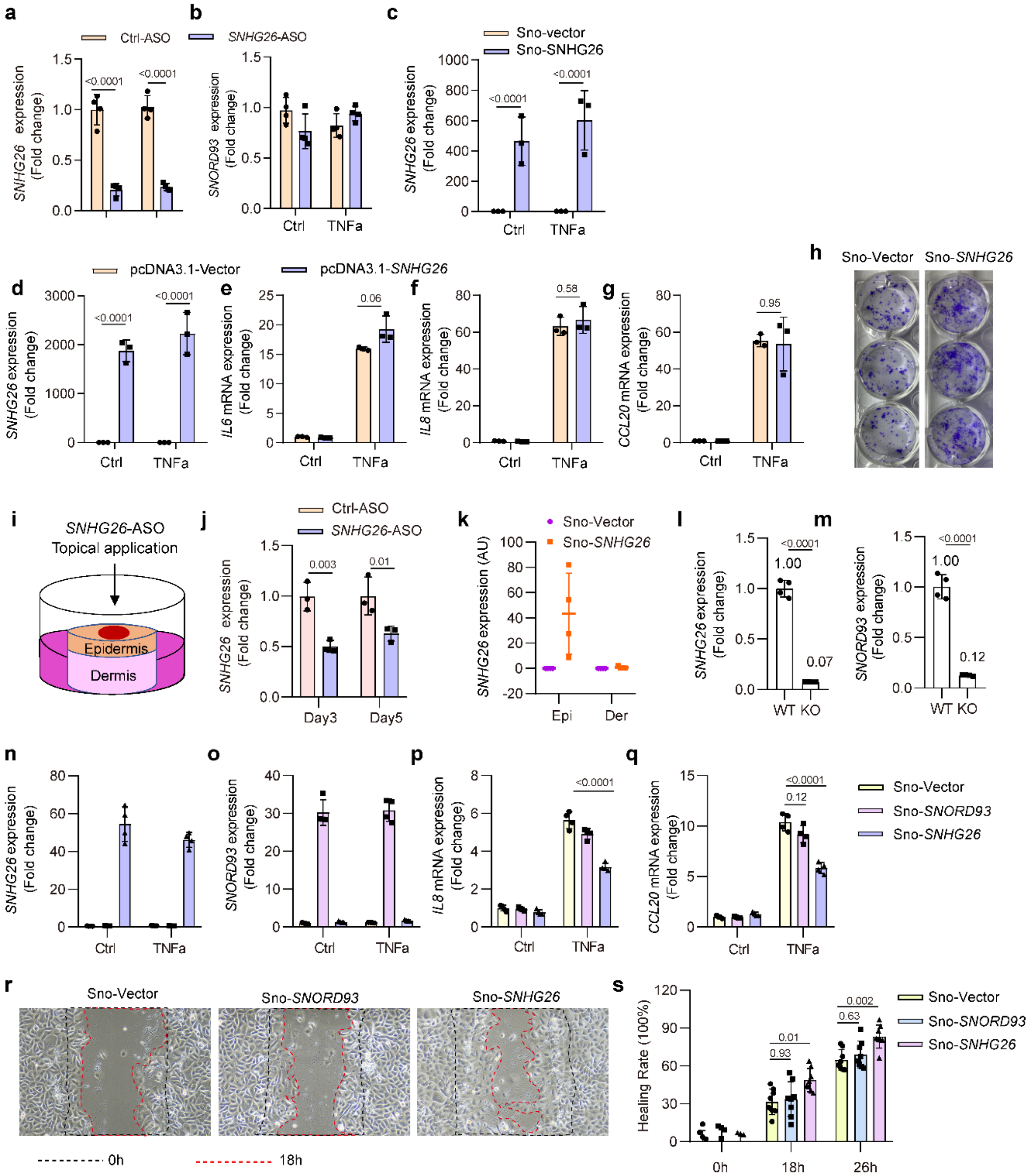


**Supplementary Figure 9.** ***SNHG26*** **inhibits the human keratinocyte inflammatory response and promotes re-epithelialization.** (**a-b**) qRT-PCR analysis of *SNHG26* and *SNORD93* in human keratinocyte progenitors with *SNHG26* knockdown followed with or not with TNFα treatment (n=3). (**c**) qRT-PCR analysis of *SNHG26* level in human keratinocyte progenitors overexpressing *SNHG26* with nuclear expression plasmid pZW1-snoVector (Sno-*SNHG26*) (n=3). (**d-g**) qRT-PCR analysis of *SNHG26*, *IL6*, *IL8*, and *CCL20* in human keratinocyte progenitors overexpressing *SNHG26* with pcDNA3.1 vector followed with or not with TNFα treatment (n=3). (**h**) Colonies formed by the Sno-Vector and Sno-*SNHG26* transfected keratinocytes were stained with crystal

violet 10 days after the transfection (n=3). (**i**) Schematic illustration of the topical application of *SNHG26*-ASOs to human *ex vivo* wounds. (**j**) qRT–PCR analysis of *SNHG26* expression in human *ex vivo* wounds with *SNHG26* knockdown (n=3). (**k**) qRT–PCR analysis of *SNHG26* expression in the epidermis (Epi) and dermis (Der) of human *ex vivo* wounds with *SNHG26* overexpression (n=4). (**l-m**) qRT-PCR analysis of *SNHG26* and *SNORD93* expression level in hTERT-immortalized human keratinocyte cell line (Ker-CT) with a knockout at the *SNHG26* locus using CRISPR-Cas9 technology (n=4). (**n-q**) *SNHG26* knockout keratinocytes were transfected with *SNORD93* or *SNHG26* overexpression plasmids (Sno-SNORD93, Sno-*SNHG26*) followed by TNFα stimulation. The expression of *SNHG26*, *SNORD93*, *IL8*, and *CCL20* was measured by qRT-PCR (n=3-4). (**r-s**) Scratch wound assay of *SNHG26* knockout keratinocytes with *SNORD93* or *SNHG26* overexpression (n=8). Data are shown as mean ± SD from two to three independent experiments. The data were analyzed by two-way ANOVA (**a**, **c-g**, **j**) or two-tailed Student's t test (**p-s**).

**Supplementary Figure 10.**

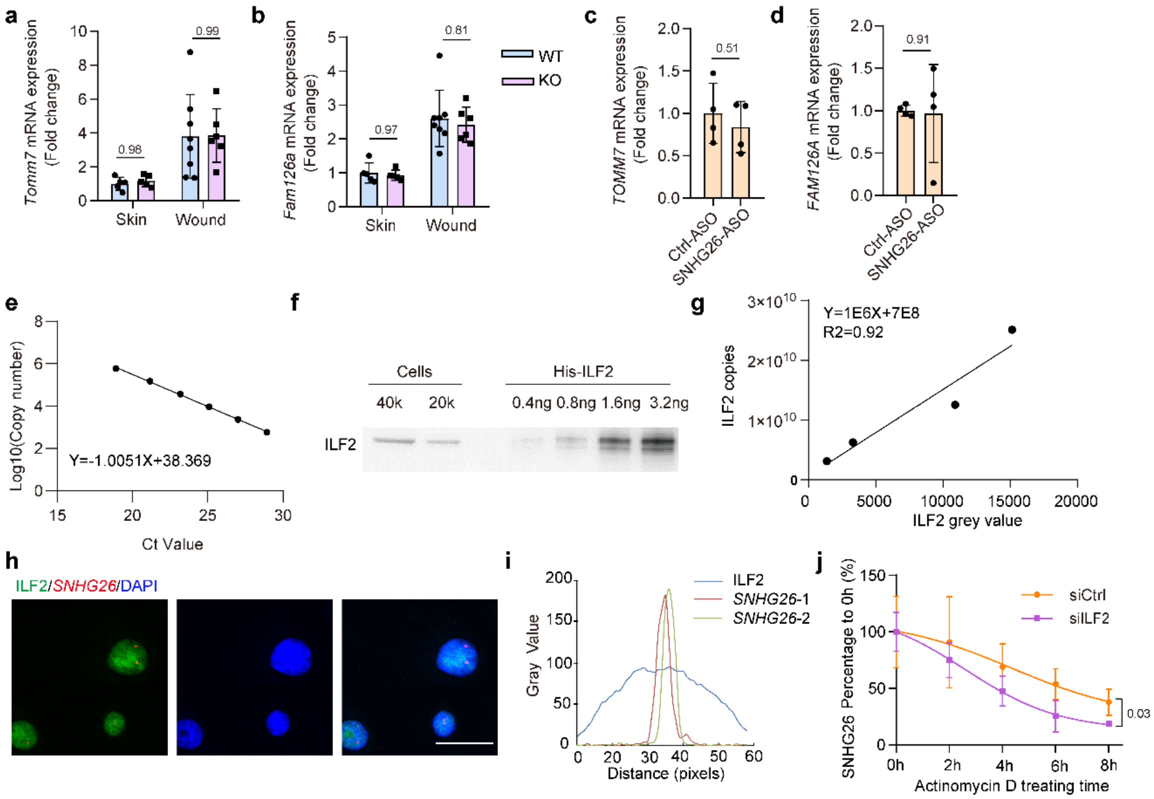


**Supplementary Figure 10. *SNHG26* interacts with ILF2.** **(a-b)** qRT-PCR analysis of *Tomm7* and *Fam126a* mRNA expression in the skin and wound of WT and SNHG26-KO mice (n=5-8). **(c-d)** qRT-PCR analysis of TOMM7 and FAM126A mRNA expression in the primary keratinocytes after SNHG26 knockdown (n=4). (**e**) To quantify the *SNHG26* transcript, we established a standard curve by amplifying a dilution series of the PZW-Sno-*SNHG26* plasmid using qPCR. Subsequently, we measured the Ct value of *SNHG26* in 50,000 keratinocytes via qPCR and calculated the copy number based on the standard curve. (**f-g**) For the quantification of ILF2 protein, we conducted ILF2 Western blotting using a dilution series of ILF2 recombinant protein in conjunction with protein lysates from 20,000 or 40,000 human keratinocytes. The standard curved was created by signal values of ILF2 immunoblot bands analyzed by Image J against the ILF2 protein molarity. The copy number of ILF2 protein in each keratinocyte was calculated by ILF2 band grey value and the standard curve. (**h**) Dual RNAscope® fluorescence in situ hybridization and immunostaining to visualize the SNHG26 RNA (red) and ILF2 protein (green) in human keratinocyte progenitors. Nuclei were stained with DAPI. Scale bar, 5 μm. (**i**) Colocalization analysis was performed with the ImageJ JACoP plugin. **(j)** Keratinocytes were transfected with ILF2 siRNA and then treated with actinomycin D (5 μg/mL) for 2-8 hours. SNHG26 expression was measured by qRT-PCR (n=3). Data are shown as mean ± SD from two to three independent experiments. The data were analyzed by two-way ANOVA (**a**, **b**, **j**) or two tailed Student's t test (**c**, **d**).

**Supplementary Figure 11.**

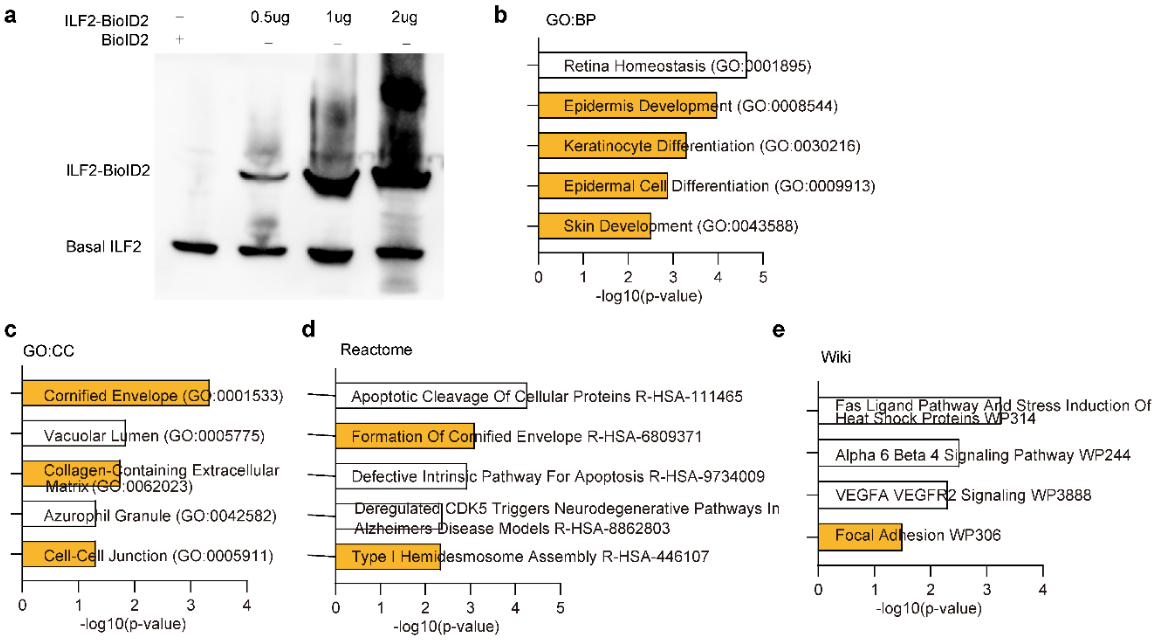


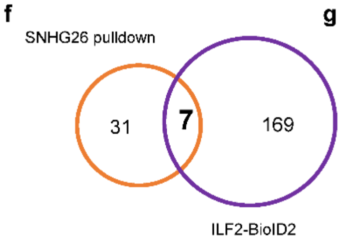


g

| Gene Symbol | Subcellular Location |
|---|---|
| ACTB | Cytosol, Nucleus |
| ANXA2 | Cytosol, Nucleus |
| LAMB3 | Vesicles |
| LAMC2 | Golgi, Cytosol, Nucleoplasm, Endoplasmic Reticulum, Vesicles. |
| LAMA3 | Endoplasmic reticulum |
| IF4A1 | Cytosol |
| TSP1 | Plasma membrane, Cytosol |

**Supplementary Figure 11. Identification of proteins proximal to ILF2 by using BioID assay.** (**a**) Western blot analysis of ILF2-BioID fusion protein in primary keratinocytes. (**b-e**) Functional enrichment analysis of proteins proximal to ILF2 in keratinocytes. **(f)** Overlap of the proteins identified by ILF2-BioID2 and SNHG26 pull down. (**g**) Subcellular location of the overlapped proteins in **f**.

**Supplementary Figure 12.**

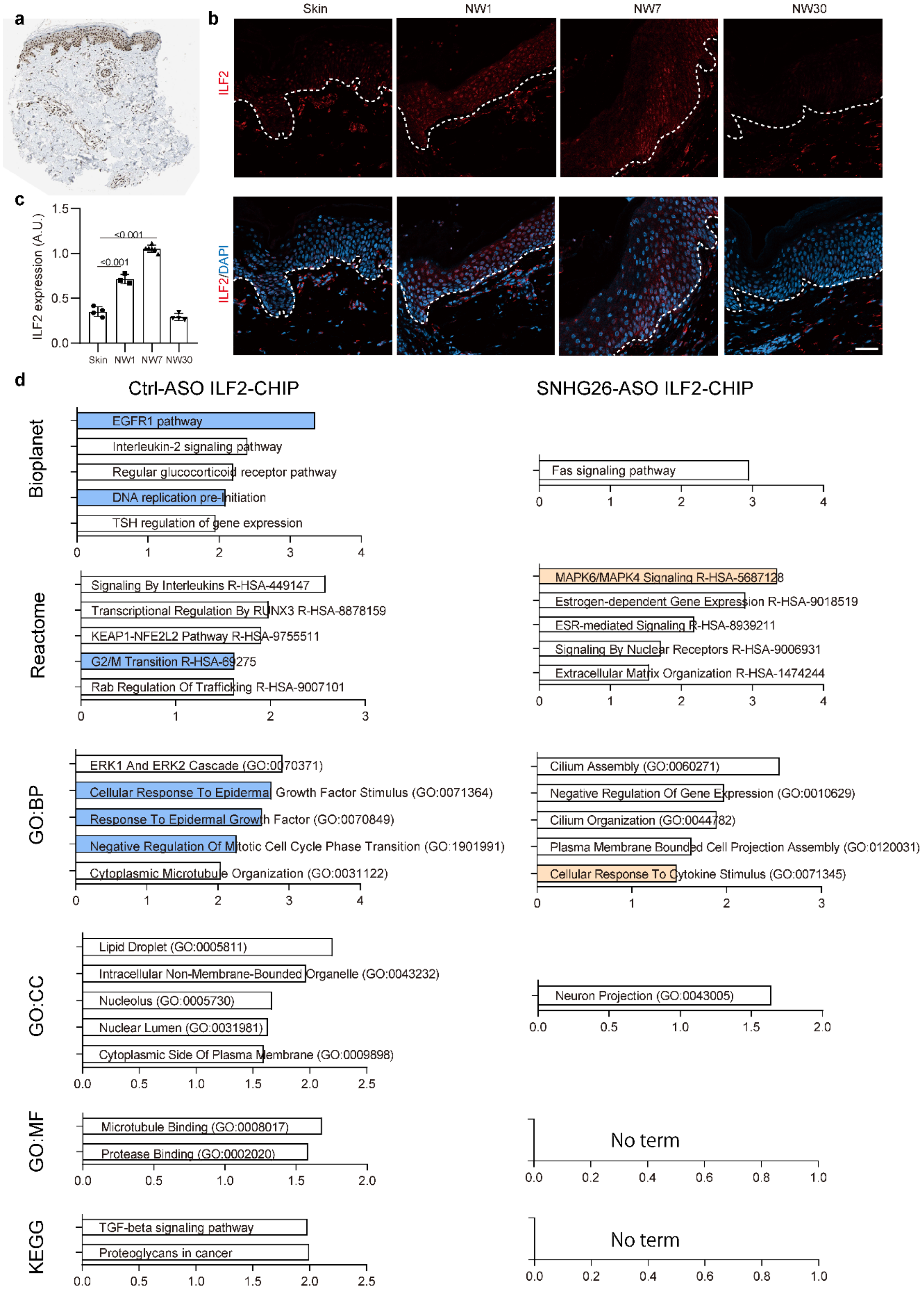

**Supplementary Figure 12. (a)** Immunohistochemistry staining of ILF2 in human skin. The picture was extracted from Human Protein Atlas (https://www.proteinatlas.org/ENSG00000143621-ILF2/tissue/skin#img). (**b**) Immunofluorescence analysis of ILF2 in human acute wounds. Scale bar, 50 μm. The white dashed line indicates the boundaries between epidermis and dermis. (**c**) Fluorescent intensity arbitrary units (AU) were analyzed by Image J (n=3-6). The data were analyzed by one-way ANOVA. (**d)** Functional enrichment analysis of the genomic loci bound by ILF2 in Ctrl-ASO and *SNHG26*-ASO transfected human keratinocyte progenitors.

**Supplementary Figure 13.**

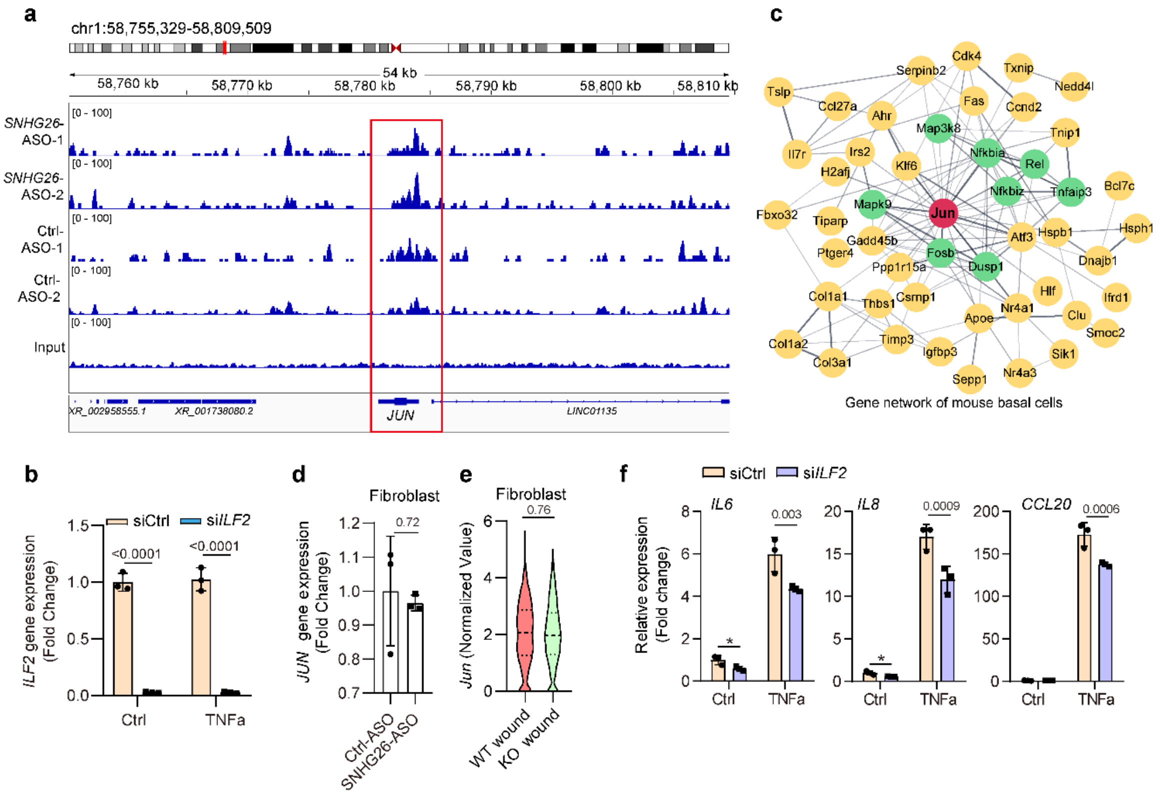


**Supplementary Figure 13. ILF2 is essential for TNFa-indued inflammatory response. (a)** Genome browser tracks of ILF2 ChIP-seq signal intensity showing increased ILF2 binding at the promoter regions of *JUN* in human keratinocyte progenitors with *SNHG26* knockdown. (**b**) qRT-PCR analysis of *ILF2* mRNA expression in human keratinocyte progenitors transfected with siRNA targeting *ILF2*(n=3). (**c**) A functional protein association network was identified among the most upregulated genes in *Snhg26*-KO mouse wound basal keratinocytes by using STRING. (**d**) qRT-PCR analysis of the expression of *JUN* in fibroblasts with *SNHG26* knockdown (n=3). (**e**) scRNA-seq analysis of *JUN* expression in fibroblasts of *Snhg26*-KO mice compared with WT mice. (**f**) qRT–PCR analysis of inflammatory gene expression in human keratinocyte progenitors with ILF2 silencing followed by TNFα stimulation (n=3). Data are shown as mean ± SD from two to three independent experiments (**b**, **f**). The data were analyzed by two-way ANOVA (**b**, **f**) or two-tailed Student's t test (**d**, **e**).

**Supplementary Figure 14.**

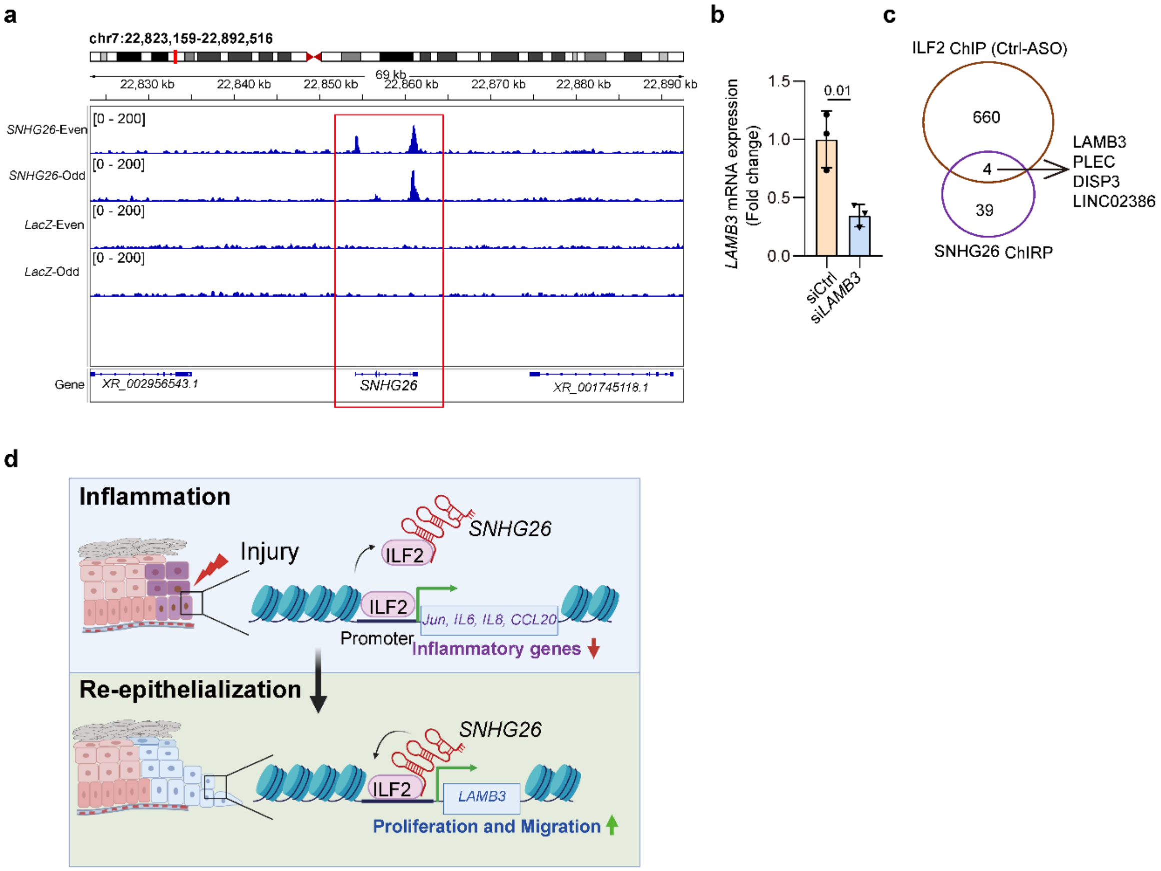


**Supplementary Figure 14.** (**a**) Genome browser tracks showing *SNHG26* occupancy at the *SNHG26* genomic locus. (**b**) qRT-PCR detection of *LAMB3* expression in human keratinocyte progenitors transfected with *LAMB3* siRNA (n=3). (**c**) Overlap of ILF2 ChIP-seq and SNHG26 ChIRP-seq results, LAMB3, PLEC, LINC02386, and DISP3 were identified as common genes bound by SNHG26 and ILF2. (**d)** Schematic showing the lncRNA SNHG26 drives the inflammatory-to-proliferative state transition of keratinocyte progenitor cells during wound healing. The data were analyzed by two-tailed Student's t test (**b**). Panel d was created with BioRender.com released under a Creative Commons Attribution-NonCommercial-NoDerivs 4.0 International license (https://creativecommons.org/licenses/by-nc-nd/4.0/deed.en).
