# Supplementary material for: The lncRNA SNHG26 drives the inflammatory-to-proliferative state transition of keratinocyte progenitor cells during wound healing
Source: Nat Commun. 2024 Oct 5;15:8637. doi: 10.1038/s41467-024-52783-8 (PMC11452505; doi:10.1038/s41467-024-52783-8)
Supplement: Supplementary file 3 — Description of Additional Supplementary Files [file 41467_2024_52783_MOESM3_ESM.pdf]

Description of Additional Supplementary Files

Supplementary Data Legends

Supplementary Data 1. Patient information

Supplementary Data 2. Differentially expressed lncRNAs in human acute wounds. (The p value was analyzed by one-way anova)

Supplementary Data 3. GO analysis of marker genes of C2, C3, and C4 clusters. The p value was analyzed by two-sided Wilcoxon test.

Supplementary Data 4. Genes used for calculating the inflammation score.

Supplementary Data 5. SNHG26 pull down MS and LAZ sequence

Supplementary Data 6. ILF2 BioID followed by MS

Supplementary Data 7. Enriched Peaks in ILF2 ChIP sequencing (Ctrl_ASO group)

Supplementary Data 8. Enriched peaks in SNHG26 ChIRP-seq

Supplementary Data 9. Oligonucleotides and Antibodies
