# Supplementary material for: The lncRNA SNHG26 drives the inflammatory-to-proliferative state transition of keratinocyte progenitor cells during wound healing
Source: Nat Commun. 2024 Oct 5;15:8637. doi: 10.1038/s41467-024-52783-8 (PMC11452505; doi:10.1038/s41467-024-52783-8)
Supplement: Supplementary file 13 — Reporting Summary [file 41467_2024_52783_MOESM13_ESM.pdf]

nature portfolio

Corresponding author(s): Dongqing Li and Ning Xu Landén

Last updated by author(s): 2024-09-02

Reporting Summary

Nature Portfolio wishes to improve the reproducibility of the work that we publish. This form provides structure for consistency and transparency in reporting. For further information on Nature Portfolio policies, see our Editorial Policies and the Editorial Policy Checklist.

Statistics

For all statistical analyses, confirm that the following items are present in the figure legend, table legend, main text, or Methods section.

n/a	Confirmed	
☐	☒	The exact sample size (*n*) for each experimental group/condition, given as a discrete number and unit of measurement
☒	☐	A statement on whether measurements were taken from distinct samples or whether the same sample was measured repeatedly
☐	☒	The statistical test(s) used AND whether they are one- or two-sided<br>*Only common tests should be described solely by name; describe more complex techniques in the Methods section.*
☒	☐	A description of all covariates tested
☒	☐	A description of any assumptions or corrections, such as tests of normality and adjustment for multiple comparisons
☐	☒	A full description of the statistical parameters including central tendency (e.g. means) or other basic estimates (e.g. regression coefficient) AND variation (e.g. standard deviation) or associated estimates of uncertainty (e.g. confidence intervals)
☒	☐	For null hypothesis testing, the test statistic (e.g. *F*, *t*, *r*) with confidence intervals, effect sizes, degrees of freedom and *P* value noted<br>*Give P values as exact values whenever suitable.*
☒	☐	For Bayesian analysis, information on the choice of priors and Markov chain Monte Carlo settings
☒	☐	For hierarchical and complex designs, identification of the appropriate level for tests and full reporting of outcomes
☒	☐	Estimates of effect sizes (e.g. Cohen's *d*, Pearson's *r*), indicating how they were calculated

Our web collection on statistics for biologists contains articles on many of the points above.

Software and code

Policy information about availability of computer code

Data collection	No software was used for data collection
Data analysis	We used the following available software: Image J (v1.0.8), GraphPad Prism 8, Cell Ranger (v5.0.1); Space Ranger (v1.2);Seurat (v4.1.0); CellChat (v1.4.0); MACS (v2.2.6); IGV_2.10.0

For manuscripts utilizing custom algorithms or software that are central to the research but not yet described in published literature, software must be made available to editors and reviewers. We strongly encourage code deposition in a community repository (e.g. GitHub). See the Nature Portfolio guidelines for submitting code & software for further information.

Data

Policy information about availability of data

All manuscripts must include a data availability statement. This statement should provide the following information, where applicable:

- Accession codes, unique identifiers, or web links for publicly available datasets
- A description of any restrictions on data availability
- For clinical datasets or third party data, please ensure that the statement adheres to our policy

The raw sequencing data have been have been deposited in the National Center for Biotechnology Information Gene Expression Omnibus (GEO) database under accession number: GSE174661 (human wound bulk RNA sequencing data, https://www.ncbi.nlm.nih.gov/geo/query/acc.cgi?acc=GSE174661), GSE216822 (microarray data of mice skin and wound epidermis, https://www.ncbi.nlm.nih.gov/geo/query/acc.cgi?acc=GSE216822), GSE216823 (microarray data of human keratinocytes transfected with SNHG26-ASO, https://www.ncbi.nlm.nih.gov/geo/query/acc.cgi?acc=GSE216823), GSE218430 (single-cell RNA sequencing data of [illegible]

mice skin and wounds, ILF2 ChIP-sequencing data and SNHG26 ChIRP sequencing data, https://www.ncbi.nlm.nih.gov/geo/query/acc.cgi?acc=GSE218430), GSE241124 (human wound spatial transcriptomic data, https://www.ncbi.nlm.nih.gov/geo/query/acc.cgi?acc= GSE241124). The mass spectrometry raw data have been deposited in the Proteomics Identification Database under the accession number PXD055231 (SNHG26 pulldown followed by mass spectrometry, https://www.ebi.ac.uk/pride/archive/projects/PXD055231) and PXD055235 (ILF2 BioID experiment, http://www.ebi.ac.uk/pride/archive/projects/PXD055235).

Research involving human participants, their data, or biological material

Policy information about studies with human participants or human data. See also policy information about sex, gender (identity/presentation), and sexual orientation and race, ethnicity and racism.

Reporting on sex and gender	The study consist 18 female and 10 male. We collected samples from all the volunteers and did not perform any gender-specific analysis.
Reporting on race, ethnicity, or other socially relevant groupings	The samples were collected from adult Caucasians. We did not select or perform analysis based on specific races, ethnicities, or other socially relevant groupings.
Population characteristics	See above.
Recruitment	The healthy volunteers were enrolled at the Karolinska University Hospital, Stockholm, Sweden. This is described in details in the methods section.
Ethics oversight	This study was approved by the Stockholm Regional Ethics Committee and conducted according to the Declaration of Helsinki's principles.

Note that full information on the approval of the study protocol must also be provided in the manuscript.

Field-specific reporting

Please select the one below that is the best fit for your research. If you are not sure, read the appropriate sections before making your selection.

☒ Life sciences ☐ Behavioural & social sciences ☐ Ecological, evolutionary & environmental sciences

For a reference copy of the document with all sections, see nature.com/documents/nr-reporting-summary-flat.pdf

Life sciences study design

All studies must disclose on these points even when the disclosure is negative.

Sample size	Sample size was determined by available subjects.
Data exclusions	Some data in the single cell RNA-seq analysis are excluded because of doublets identified by Scrublet. We also removed mitochondrial genes, hemoglobin genes, ribosomal genes, genes expressed in less than 10 cells, as well as cells with less than 500 detected genes, less than 1000 UMIs and with more than 10% mitochondrial gene expression.
Replication	All animal and cell experiments were successfully replicated at least in 2-3 independent experiments.
Randomization	Wild-type mice were randomly allocated to control or model groups. Similarly, cells were randomly distributed across different groups, and the analyses were conducted independently.
Blinding	The investigators were blinded during data collecting and analysis.

Reporting for specific materials, systems and methods

We require information from authors about some types of materials, experimental systems and methods used in many studies. Here, indicate whether each material, system or method listed is relevant to your study. If you are not sure if a list item applies to your research, read the appropriate section before selecting a response.

Materials & experimental systems

n/a	Involved in the study	
☐	☒	Antibodies
☐	☒	Eukaryotic cell lines
☒	☐	Palaeontology and archaeology
☐	☒	Animals and other organisms
☒	☐	Clinical data
☒	☐	Dual use research of concern
☒	☐	Plants

Methods

n/a	Involved in the study	
☐	☒	ChIP-seq
☒	☐	Flow cytometry
☒	☐	MRI-based neuroimaging

Antibodies

Antibodies used

Mouse monoclonal anti human ILF2 (Santa Cruz, sc-365283, 1:500); Rat monoclonal anti mouse CD68 (FA-11) (Bio-Rad Laboratories, MCA1957, 1:500); JUN Monoclonal antibody (Proteintech, 66313-1-Ig, 1:1000)

Validation

Antibody reactivates are validated by the literature or supplier as follows:
ILF2: https://doi.org/10.1371/journal.pone.0216042.
CD68: https://www.bio-rad-antibodies.com/monoclonal/mouse-cd68-antibody-fa-11-mca1957.html?f=purified.
JUN: https://www.ptglab.com/products/JUN-Antibody-66313-1-Ig.htm.

Eukaryotic cell lines

Policy information about cell lines and Sex and Gender in Research

Cell line source(s)

Human adult primary keratinocytes (C0055C; Thermo Fisher) were cultured in Epilife medium supplement with Human Keratinocyte Growth Supplement (HKGS, S0015, Thermo Fisher) and 1X Penicillin-Streptomycin.

Authentication

HEKa were obtained from Thermo Fisher

Mycoplasma contamination

The cells tested negative for Mycoplasma.

Commonly misidentified lines
(See ICLAC register)

N/A

Animals and other research organisms

Policy information about studies involving animals; ARRIVE guidelines recommended for reporting animal research, and Sex and Gender in Research

Laboratory animals

The C57BL/6J wild type mice were obtained from Charles River Laboratories (Sulzfeld, Germany). The Snhg26 knockout mice on C57BL/6J background and their control littermates used in this study were generated by Cyagen Biosciences Company (Santa Clara, US).All the mice were breed under pathogen-free conditions in Comparative Medicine Biomedicum (KMB) animal facility at Karolinska Institutet under standard laboratory conditions, which included free access to food and water, a 12-hour light/dark cycle, a stable temperature range of 20-22°C, and humidity maintained between 40-60%. The 8–10 weeks old mice with C57BL/6J background were used in all the experiments.

Wild animals

No wild animals are used.

Reporting on sex

Both male and female mice were used.

Field-collected samples

N/A

Ethics oversight

All the mice were breed under pathogen-free conditions in Comparative Medicine Biomedicum (KMB) animal facility at Karolinska Institutet. All the mouse experiments were approved by committee on animal experimentation of Swedish Board of Agriculture (Jordbruksverket).

Note that full information on the approval of the study protocol must also be provided in the manuscript.

Plants

Seed stocks	N/A
Novel plant genotypes	N/A
Authentication	N/A

ChIP-seq

Data deposition

☒ Confirm that both raw and final processed data have been deposited in a public database such as GEO.

☒ Confirm that you have deposited or provided access to graph files (e.g. BED files) for the called peaks.

Data access links<br>*May remain private before publication.*	https://www.ncbi.nlm.nih.gov/geo/query/acc.cgi?acc=GSE218430
Files in database submission	All files available will be listed on https://www.ncbi.nlm.nih.gov/geo/query/acc.cgi?acc=GSE218430
Genome browser session<br>(e.g. UCSC)	N/A

Methodology

Replicates	ChIP-Seq data were obtained from 2 replicate samples per group. ChIP validation by qPCR of prioritized gene targets were obtained from 3 replicate samples per group.
Sequencing depth	The sequencing was performed on DNBSEQ-G400 platform with PE100. The sequencing depth is 30 Million reads.
Antibodies	Mouse monoclonal anti human ILF2 (Santa Cruz, sc-365283)
Peak calling parameters	Peaks were called using MACS2 (v2.2.6) with the default parameter
Data quality	Raw sequencing data were processed by using Trimmomatic v0.36 to trim reads of low quality and shorter than 20 nucleotides.
Software	Bowtie2, Picard,MACS2 (v2.2.6), Trimmomatic v0.36.
